# Dorsal Striatal Dopamine, Food Preference and Health Perception in Humans

**DOI:** 10.1371/journal.pone.0096319

**Published:** 2014-05-07

**Authors:** Deanna L. Wallace, Esther Aarts, Linh C. Dang, Stephanie M. Greer, William J. Jagust, Mark D′Esposito

**Affiliations:** 1 Helen Wills Neuroscience Institute, University of California, Berkeley, California, United States of America; 2 Center for Cognitive Neuroimaging, Donders Institute for Brain, Cognition and Behaviour, Radboud University, Nijmegen, The Netherlands; 3 Department of Psychology, Vanderbilt University, Nashville, Tennessee, United States of America; University of Western Ontario, Canada

## Abstract

To date, few studies have explored the neurochemical mechanisms supporting individual differences in food preference in humans. Here we investigate how dorsal striatal dopamine, as measured by the positron emission tomography (PET) tracer [^18^F]fluorometatyrosine (FMT), correlates with food-related decision-making, as well as body mass index (BMI) in 16 healthy-weight to moderately obese individuals. We find that lower PET FMT dopamine synthesis binding potential correlates with higher BMI, greater preference for perceived “healthy” foods, but also greater healthiness ratings for food items. These findings further substantiate the role of dorsal striatal dopamine in food-related behaviors and shed light on the complexity of individual differences in food preference.

## Introduction

Modern society is surrounded by an overabundance and a wide-variety of food choices, which in part contributes to the growing overweight population in the United States [1]. Yet, the underlying neurochemical mechanisms supporting individual differences in food preferences are not well-understood. Some individuals naturally base their food preferences more on the health value of food items versus the taste value of food items, and the ventromedial prefrontal cortex (vmPFC) has been shown to play a role in goal values related to influences of “health” and “taste” [2]. Furthermore, there is a wide variation in individuals' judgment of caloric content and perceived “healthiness” of food items [3], and studies show perceived “healthy” foods are over-consumed as compared to perceived “unhealthy” foods, despite equal nutritional value [3], [4].

Dorsal striatal dopamine has been shown to play a role in motivation for food in both human and animal models [5], [6], [7], yet the relationship between dopamine and food desirability or preferences in humans has not been thoroughly explored. Additionally, studies that utilize PET ligands that bind dopamine receptors have shown correlations with BMI, however, in both positive [8] and negative [9] directions, and not all studies find significant associations (for review see [10]). Also, due to the nature of these PET ligands that are dependent on the state of endogenous dopamine release, it is difficult to interpret relationships between striatal dopamine and BMI. Lower dopamine receptor binding could represent fewer existing striatal dopamine receptors (i.e. a negative relationship between PET binding and BMI, as found in [9]), or greater dopamine receptor binding could represent lower endogenous dopamine release, allowing more available receptors in which the PET ligand could bind (i.e. a positive relationship between binding and BMI, as found in [8]). To complement previous studies that have utilized PET ligands that bind dopamine receptors, here we used a stable measurement of presynaptic dopamine synthesis capacity with the PET ligand [^18^F]fluorometatyrosine (FMT) that has been extensively studied in human and animal models [11], [12], [13], [14].

The aims of our study were to investigate the relationship between dorsal striatal PET FMT dopamine synthesis measures and BMI and to study how these PET FMT dopamine synthesis measures may correlate with individual differences in food preference. We hypothesized that lower PET FMT dopamine synthesis binding would correspond with higher BMI, as suggested by previous work [15]. We also predicted that individuals with lower endogenous striatal dopamine would have greater overall preference for food items (i.e. both “healthy” and “unhealthy” foods) as compared to individuals with higher striatal dopamine and that an individual's health perception of food items may also influence preference.

## Methods and Materials

### Subjects

Thirty-three healthy, right-handed subjects who previously received PET FMT dopamine synthesis scans were invited to participate in the behavioral study presented here and were given no prior knowledge to the study, only informed that it involved studying complex decision-making. Of these 33, 16 subjects agreed to participate (8 M, age 20–30). BMI ((weight in kilograms)/(height in meters)∧2)) was calculated for all subjects (range: 20.2–33.4, with 1 obese, 4 overweight and 11 healthy-weight subjects). Subjects had no history of drug abuse, eating disorders, major depression and anxiety disorders. Subjects were also asked to if they were in very poor, poor, average, good or excellent health. All reported to be in overall average to excellent health and not currently dieting or trying to lose weight. Socioeconomic status (SES) was also collected from individuals using the Barratt simplified measure of social status (BSMSS) [16].

### Ethics Statement

All subjects gave written informed consent and were paid for participation according to institutional guidelines of the local ethics committee (University of California Berkeley (UCB) and Lawrence Berkeley National Laboratory (LBNL) Committee for the Protection of Human Participants (CPHP) and Lawrence Berkeley National Laboratory Institutional Review Boards (IRB)). UCB's and LBNL's CPHPs and IRBs specifically approved the studies presented here

### PET data acquisition and analysis

PET imaging and FMT binding were performed at Lawrence Berkeley National Laboratory, as described previously [17]. FMT is a substrate of aromatic L-amino acid decarboxylase (AADC), a dopamine-synthesizing enzyme whose activity corresponds to the capacity of dopaminergic neurons to synthesize dopamine [13] and has been shown to be indicative of pre-synaptic dopamine synthesis capacity [18]. FMT is metabolized by AADC to [^18^F]fluorometatyramine, which is oxidized to [^18^F]fluorohydroxyphenylacetic acid (FPAC), remains in the dopaminergic terminals and is visible on PET FMT scans. Thus, signal intensity on PET FMT scans has been shown to be comparable with [^18^F]fluorodopa [18], in which tracer uptake is highly correlated (r = 0.97, p<0.003) with striatal dopamine protein levels in post-mortem patients, as measured by high performance liquid chromatographic (HPLC) methods [19]. Moreover, in comparison to [^18^F]fluorodopa, FMT is also not a substrate for O-methylation and therefore provides higher signal-to-noise images than [^18^F]fluorodopa [18]. Additionally, FMT measures have been shown to directly correspond with dopamine measures in animal Parkinson's disease models [14].

Scans were conducted either from 9AM-12PM or 1PM-4PM. The average delay between acquisition of the PET FMT dopamine synthesis data and the behavioral data was 2.37±0.26 years, comparable to the delay reported in a previous study from our lab utilizing PET FMT [11]. Although this delay is not ideal, a study by Vingerhoets et al. [20] has shown that striatal Ki related to presynaptic dopamine is a relatively stable measurement, having a 95% chance of remaining within 18% of its original value within individual healthy subjects over a 7-year time-span. Therefore, FMT measures, comparable to [^18^F]fluorodopa [13], are thought to reflect relatively stable processes (i.e. synthesis capacity) and therefore not particularly sensitive to small state-related changes. Additionally, BMI was not significantly different between the acquisition of the PET and behavioral data (average change in BMI: 0.13±1.45, T(15) = 0.2616, p = 0.79, two-tailed paired t-test). Also, all subjects were screened for any lifestyle changes in the time since last testing (i.e. change in diet and exercise/daily activity, smoking or drinking, mental health or medication status). Finally, change in BMI from time of the PET FMT scan to behavioral testing as well as the time elapsed between PET scan and behavioral testing were used as variables in the multiple regression data analysis.

PET scans were performed using the Siemens ECAT-HR PET camera (Knoxville, TN). Approximately 2.5 mCi of high specific activity FMT was injected as a bolus into an antecubital vein and a dynamic acquisition sequence in 3D mode was obtained for a total of 89 min scan time. Two high-resolution anatomical images (MPRAGE) were acquired in each participant on a Siemens 1.5 T Magnetom Avanto MRI scanner (Siemens, Erlangen, Germany), using a 12-channel head coil (TE/TR = 3.58/2120 ms; voxel size = 1.0×1.0×1.0 mm, 160 axial slices; FOV = 256 mm; scanning time ∼9 minutes). The two MPRAGEs were averaged to obtain one high-resolution structural image, which was used to generate individual caudate and cerebellum regions of interest (ROI).

Left and right caudate and cerebellum ROIs (used as reference region, as in previous studies [11]) were manually drawn on each participant's anatomical MRI scan using FSLView (http://www.fmrib.ox.ac.uk/fsl/), as described previously [21]. Both inter- and intra-rater reliability were above 95% (from ratings made by two lab members). To avoid contamination of FMT signal from dopaminergic nuclei, only the posterior three-fourths of the gray matter were included in the cerebellar reference region. After co-registration to PET FMT space, only the voxels with an above 50% chance to lie in the ROIs were included to ensure high grey matter probability.

PET FMT images were reconstructed with an ordered subset expectation maximization algorithm with weighted attenuation, scatter corrected, motion-corrected and smoothed with a 4 mm full width half maximum kernel, using Statistical Parametric Mapping version 8 (SPM8) (www.fil.ion.ucl.ac.uk/spm/). The anatomical MRI scan was coregistered to the mean image of all realigned frames in the PET FMT scan using FSL-FLIRT (http://www.fmrib.ox.ac.uk/fsl/, version 4.1.2). Using an in-house graphical analysis program implementing Patlak plotting [22], [23], K_i_ images, representing the amount of tracer accumulated in the brain relative to the reference region (cerebellum [11], , a standard practice in PET analysis to minimize potential confounds of noise from PET data), were created. K_i_ values were obtained separately from the left and right caudate ROIs and associations were computed between K_i_ values, BMI, and the behavioral measures. Additionally, since age and sex have been shown to have an effect on FMT binding [15], [24], correlations between FMT and BMI were corrected for age and sex (as well as any changes in BMI from time of PET scan to behavioral testing) by control variables in a Pearson's partial correlation.

### Behavioral paradigm

Subjects were asked to eat a typical, but not too heavy meal an hour prior to the testing session. In order to encourage compliance with this request, testing sessions were scheduled after typical meal times (i.e. 9AM, 2PM and 7:30PM), and time of last meal was recorded. Food items consumed prior to testing and the elapsed time from last meal eaten to testing session were recorded, (as determined by the resource www.caloriecount.com and meal and serving sizes self-reported by individual). To ensure hunger was not influencing the task, we also measured hunger and fullness with a visual analog scale [25].

**Figure 1:**
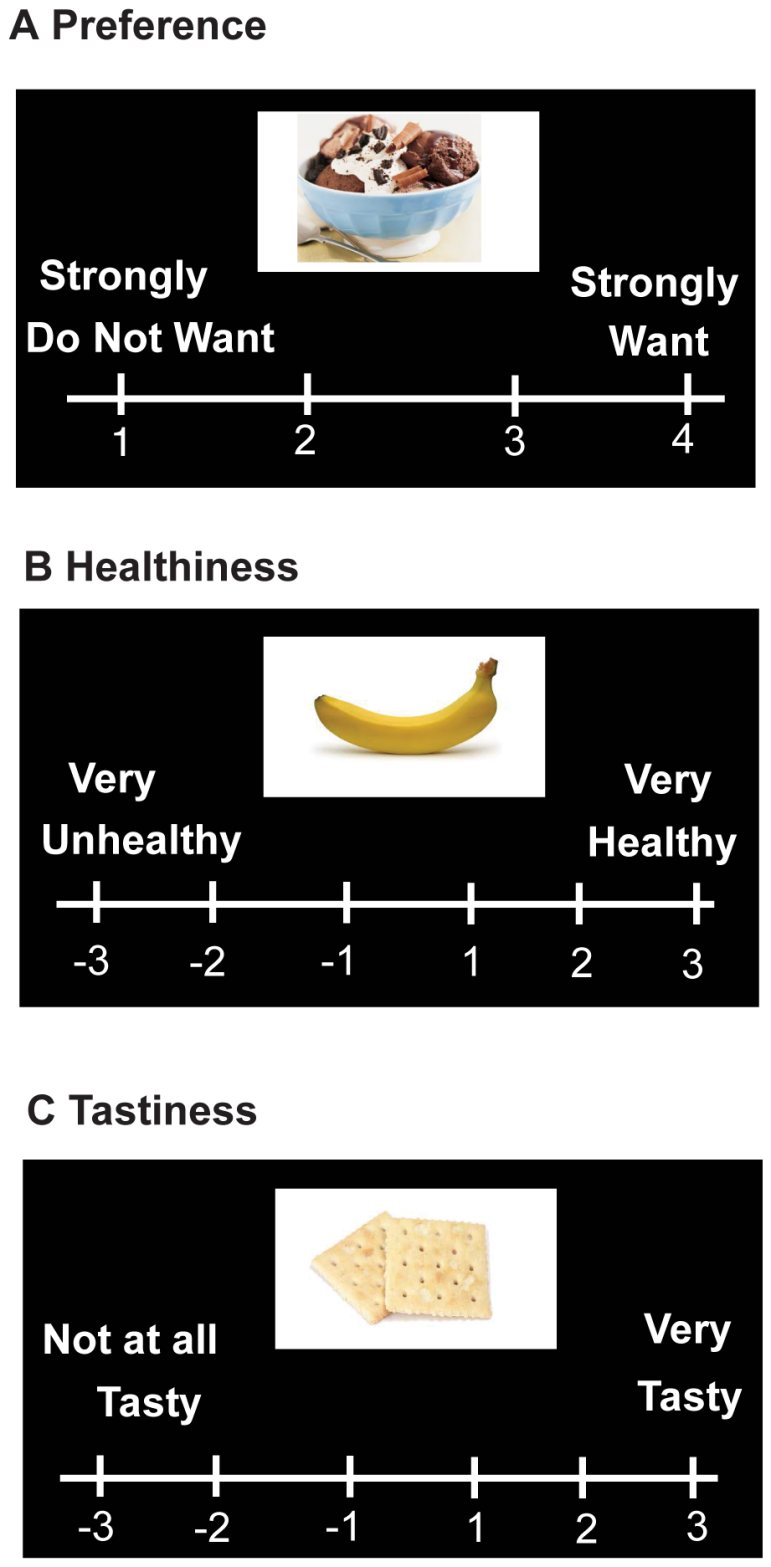
Behavioral Task. Subjects rated food items based on **A**) Preference (i.e. “wanting/desirability”), rated on a scale of 1–4 **B**) Perceived healthiness, rated on a scale of −3 to +3 and **C**) Tastiness, rated on scales −3 to +3. Food items rated 3 or 4 in the preference/desirability block were scored as “preferred”, while those rated 2 or 3 in the healthiness/tastiness blocks were rated as perceived “healthy”/“tasty” and −2 or −3 as perceived “unhealthy”/“untasty”.

Pictures of eighty food items were used in which subjects were asked to rate the items in 3 separate blocks based on 1) desirability, 2) healthiness and 3) tastiness in the program E-Prime Professional (Psychology Software Tool, Inc., Sharpsburg, PA, USA) (see **Figure 1**). In order to create a task with balanced numbers of healthy, unhealthy and neutral food items, we first created an objective health value for each of the eighty food items by assigning a standardized, objective score of -3 (very unhealthy) to +3 (very healthy) to each food based on a letter grade (ranging from F-minus (very unhealthy) to A-plus (very healthy)) and nutritional information from the on-line resource www.caloriecount.com. These letter grades incorporate several factors (i.e. calories, grams of fat, fiber etc.) and are listed as an on-line reference for “choices for healthy eating,” as stated on the website. We then balanced the task with approximately equal numbers of healthy (i.e. foods with objective scores of 2 or 3, such as fruit and vegetables), neutral (i.e. foods with objective scores of 1 and −1, such as saltine crackers) and unhealthy items (i.e. foods with negative objective scores of −2 or −3 such as highly processed candy bars).

Subjects were first asked to rate the degree to which they “desired” or “wanted” each item (scale of 1 (strongly do not want) to 4 (strongly want)), referred to throughout the text as “preferred”, a term consistent with the literature [2]. The food item would appear and the subject would have up to 4 seconds to respond, and they rated all eighty food items before continuing to the subsequent “health” and “taste” blocks (see below). Because humans have the capacity to modulate food choices based not only on taste for certain foods, but also on perceptions of healthiness [26], we only asked the subject to rate how much they would want the food or find the food desirable and the preference block was always presented first. In an attempt to capture how much the subject actually preferred the food items presented, subjects were informed they would receive a food item from the task at the end of testing based on their “desirability” ratings. The subjects also did not know in the upcoming second and third blocks (described below), they would be asked to judge how healthy and tasty they found each food item.

In the second block, subjects rated how much they perceived the eighty food items as healthy or unhealthy (−3 for very unhealthy to 3 for very healthy) and in a third block, how tasty they found the eighty food items (−3 for not at all tasty to 3 for very tasty). The order of these blocks was consistent for all subjects, as we did not want to influence health ratings in a potential order effect. The subjects were informed that the ratings of health and taste would not affect the item they would receive based on their answers in the “desirability” block. We chose a 6-point scale for health and taste values to allow a wider range of measuring taste/health perception, including a “neutral” rating corresponding to −1 and +1, whereas the 4-point scale of the desirability/preference block would reflect only preferred or non-preferred food items. The total task lasted approximately 25 minutes. Subjects were asked at the end of the task if there were any food items that were unfamiliar that may have led to non-responses. All subjects reported familiarity with food items and all items were given ratings for all three blocks by all subjects.

Dopamine in the dorsal striatum has been shown to have a strong association in motivation for food [5], [6], [7]. Taste perception is also highly correlated with desirability of food, in that most humans prefer foods that they also find tasty [27]. Because there are many combinations of the preference, taste and health blocks that could be examined, to eliminate multiple comparisons and the potential for spurious correlations, based on this literature, we examined the number of food items that were self-rated as 1) preferred, tasty, and perceived “healthy” and 2) preferred, tasty, and perceived “unhealthy”. (Preferred items rated as 3 or 4 in the “desirability” block; tasty items rated as 2 or 3 in the “tastiness” block; perceived “healthy” items rated as 2 or 3 and perceived “unhealthy” items rated as −2 or −3 in the “healthiness” block). Post-hoc analysis also investigated the ratio of perceived “healthy”-to-“unhealthy” food items, the number of preferred perceived “healthy” food items that were not actually objectively rated as healthy (i.e preferred items that the individual rated as healthy minus items the subject rated as preferred that were actually healthy as determined by the assigned objective health score. (For example, if a subject rated “crackers” as a preferred perceived healthy food with a healthy score of 3 (very healthy), and the assigned objective health score was a 1 (neutral-healthy), this would be counted as a preferred perceived healthy food that was not actually healthy). Average calories for preferred items from each individual subject were also calculated.

### Statistical Analysis

Step-wise multiple linear regression was used to test the relationships between the two separate dependent variables: 1) preferred, tasty and perceived healthy and 2) preferred, tasty and perceived unhealthy food items, and the independent variables: right caudate PET FMT values, left caudate PET FMT values, BMI, age, sex, socio-economic status, any changes in BMI between PET and behavioral testing and time elapsed between PET and behavioral testing in SPSS version 19 (IBM, Chicago, Ill., USA), with inclusion of the independent variable to the model set at p<0.05 and excluded with p>0.1. The perceived “healthy”-to-“unhealthy” ratio was highly correlated with the dependent variable of preferred perceived “healthy” items (r = 0.685, p<0.003), and therefore, we were unable to enter this variable into the model. However, Pearson's partial correlations, corrected for age, sex and any BMI changes, were used to test direct relationships between right caudate PET FMT and 1) BMI, 2) perceived “healthy”-to-“unhealthy” ratio and 3) average calories of preferred items, conducted with SPSS version 19 (IBM, Chicago, Ill., USA). We also further tested the relationship between PET FMT dopamine synthesis values, the number of preferred perceived “healthy” food items that were not rated as healthy by the calculated score, and preferred items that were rated as healthy by the calculated score in a step-wise multiple regression model. (The number of preferred perceived “healthy” food items not rated as healthy by the calculated score, and preferred items rated as healthy by the calculated score were not significantly correlated (r = 0.354, p = 0.23). We also tested if there was a relationship between change in BMI and the dependent variables: left and right caudate PET FMT values, SES, age, sex, time between PET imaging and behavioral testing, number of preferred perceived “healthy” foods and preferred perceived “unhealthy” foods using step-wise linear regression. Data are shown as Pearson r-values.

## Results

### Relationship between PET FMT dopamine synthesis values and BMI

**Figure 2:**
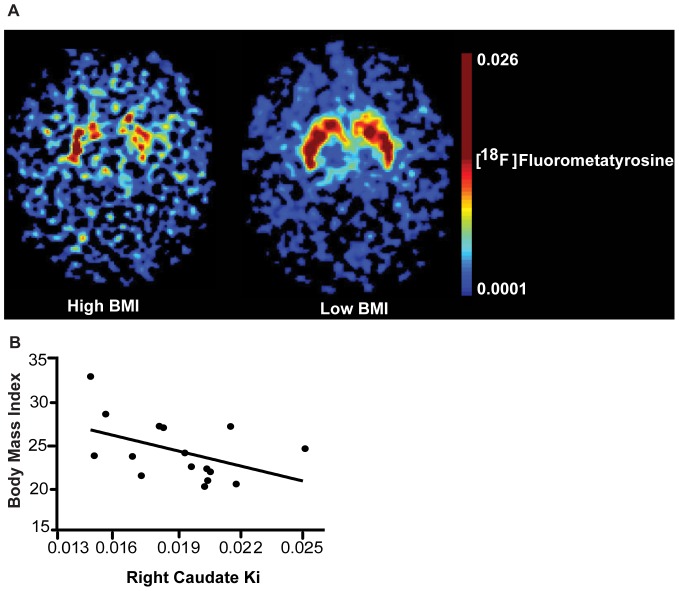
Dorsal striatal dopamine and BMI. **A**) PET imaging with FMT showed lower dorsal striatal dopamine synthesis capacity in a representative higher BMI individual (left) than a representative lower BMI individual (right, raw images for illustration purposes only). **B**) BMI and dorsal striatal dopamine were negatively correlated as measured by the PET ligand FMT, a measure of presynaptic dopamine synthesis capacity, relative to the cerebellum (r = −0.66, p = 0.014, n = 16, controlled for age, sex and any changes in BMI from PET scan to behavioral testing).

We first tested whether a significant relationship exists between caudate PET FMT dopamine synthesis values and BMI measurements across 16 individuals (average-to-moderately overweight/obese individuals). We found a significant negative correlation between caudate PET FMT dopamine synthesis values and BMI, with higher BMI individuals having lower dopamine synthesis (**Figure 2A**: PET FMT raw images of higher (left) and lower (right) BMI individuals; **Figure 2B**: right caudate, r = −0.66, p = 0.014, left caudate: r = −0.22, p = 0.46 (not significant (n.s.)), controlled for age, sex and any changes in BMI from PET FMT dopamine synthesis scan to behavioral testing).

### Relationship between PET FMT dopamine synthesis values and food preference

Subjects rated eighty food items in 3 separate blocks based on the their perception of 1) desirability, 2) healthiness and 3) tastiness of each food item (see **Figure 1**). Approximately 50% of the items were healthy and unhealthy, as set forth by health information (See **Methods and Materials**). Dopamine in the dorsal striatum has been shown to have a strong association in motivation for food [5], [6], [7], while hedonic properties of food are mediated through other neuronal mechanisms [7], [27]. However, taste perception is highly correlated with desirability of food, in that most humans prefer foods that they also find tasty [27]. Here we also find that taste perception and preference are highly correlated, in that items preferred are also rated as tasty (r = 0.707, p<0.002).

**Figure 3:**
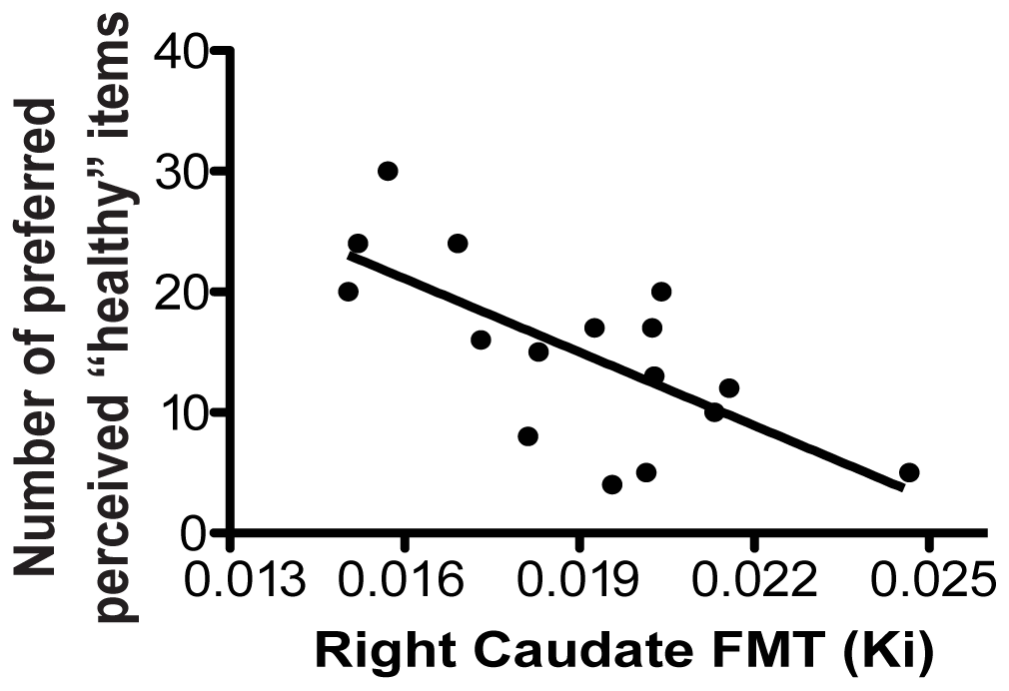
Dorsal striatal dopamine and food-related behaviors. A significant relationship was found between lower caudate PET FMT dopamine synthesis values and greater preference for perceived “healthy” food items (Beta:−0.696; t(15) = −3.625, p<0.003).

Therefore, to examine how health perception may influence food-related decision-making, we utilized step-wise multiple linear regression to model the relationships between the dependent variable of the number of food items rated as preferred, tasty and perceived healthy and the independent variables of FMT in the left and right caudate, BMI, age, sex, SES, change in BMI from time of PET scan to behavioral testing and time elapsed from time of PET to behavioral testing. Right caudate PET FMT dopamine synthesis values significantly contribute to the regression model for the number of preferred, tasty items that were perceived as healthy (Beta:−0.696; t(15) = −3.625, p<0.003, **Figure 3**), while all other independent variables were excluded from the model as non-significant (t(15)<1.216, p>0.246). We also tested the hypothesis that the number of preferred, perceived “unhealthy” items would also show a relationship with these independent variables, but no independent variable was entered into the model as significant (F<2.7, p>0.1). Thus, individuals with lower caudate PET FMT dopamine synthesis values have greater preferences for perceived “healthy” but not perceived “unhealthy” food items.

### Relationship between PET FMT dopamine synthesis values and health perception of food items

We hypothesized that the relationship between caudate PET FMT dopamine synthesis values and preference for perceived “healthy” items may be due to individual differences in the health perception of food items. Although we designed the task with an approximate 1∶1 ratio of healthy to unhealthy food items, individuals varied widely in their perception of the healthiness of the items, with ratios of healthy to unhealthy items ranging from 1.83∶1 to 0.15∶1. Therefore, as a post-hoc analysis, we investigated the relationship between right caudate PET FMT dopamine synthesis and the ratio of perceived “healthy” to “unhealthy” items, and found a significant negative correlation (r = −0.534, p = 0.04), with lower caudate PET FMT dopamine synthesis values corresponding to greater numbers of items perceived as “healthy” compared to “unhealthy”.

We therefore utilized step-wise multiple linear regression to investigate the relationships between caudate PET FMT dopamine synthesis and preference for perceived healthy but not actual healthy foods (as determined by the objective calculated score, see **Methods**), and preference for healthy foods as determined by the objective calculated score. We found a significant relationship between caudate PET FMT dopamine synthesis values and preference for perceived healthy but not actual healthy foods (Beta: −0.631, t(15) = −3.043, p<0.01), but no significant relationship between caudate PET FMT dopamine synthesis values and preference for actual calculated healthy foods (t(15) = −1.54, p>0.148), indicating preference for over-perceived “healthy” foods correlated more strongly in lower FMT individuals. Furthermore, there was no significant relationship between caudate PET FMT dopamine synthesis values and the average calories of preferred items (r = 0.288, p>0.34), indicating that lower PET FMT dopamine synthesis individuals did not differ in the caloric content of preferred foods.

We also did not find any relationship between change in BMI and PET FMT dopamine synthesis values, SES, age, sex, time between PET imaging and behavioral testing, number of preferred perceived “healthy” foods or preferred perceived “unhealthy” foods (p>0.1).

Time of testing session, time elapsed since last meal, and number of calories eaten at the last meal were not significantly correlated with any behavioral measures (p>0.13). Hunger and fullness measures also did not correlate with any of the behavioral measures (p>0.26).

## Discussion

The aim of this study was to investigate the relationship between endogenous caudate dopamine synthesis, BMI and food-related behavior. We found that lower caudate dopamine synthesis as measured by PET FMT dopamine synthesis correlated with 1) greater BMI and 2) greater preference for perceived “healthy” foods. We also found a relationship between lower caudate PET FMT dopamine synthesis values and greater over-rating of the healthiness of food items, as well as a significant correlation with greater preferred perceived “healthy” foods that were not actually healthy. We found no significant relationship between PET FMT dopamine synthesis and the average caloric content of preferred food items.

Research suggests that preference for and overconsumption of unhealthy foods are two of the many contributors to weight gain and higher BMI (Centers for Disease Control and Prevention; http://www.cdc.gov/obesity/index.html). Interestingly, we found lower dorsal striatal dopamine synthesis correlated with greater numbers of preferred, perceived “healthy” food items. Although this correlation cannot imply causation, this finding suggests endogenous differences in dorsal striatal dopamine synthesis may in part play a role in individual differences for food preference. Here we propose that lower caudate PET FMT dopamine synthesis values represent lower tonic dopamine, which in response to palatable stimuli, allows for greater phasic bursting [28] and perhaps altered responsivity to foods. Additionally, these differences in dorsal striatal dopamine may affect processing of gustatory stimuli in somatosensory cortex, as a previous study has shown altered activation in both dorsal striatal and somotosensory regions with food intake in individuals susceptible to obesity [29]. Lower dorsal striatal dopamine may also result in connectivity differences between the dorsal striatum and dorsolateral prefrontal cortex (DLPFC), as suggested by our recent findings [30]. Therefore, we hypothesize dopamine-related dorsal striatal mechanisms may influence health perception differences through either connectivity with somatosensory processing (i.e. altered taste sensation properties) or perhaps connectivity with DLPFC, which has been shown to play a role in over-evaluation of previously preferred choice items [31]. Functional magnetic resonance imaging (fMRI) could elucidate these potential mechanisms of individual differences in food preferences and over-rating of health values.

Initially, we predicted that individuals with lower dorsal striatal dopamine would have greater overall food preference (i.e. prefer more number of items self-rated as “healthy” and “unhealthy”), as compared to individuals with higher dorsal striatal dopamine. However, another finding of our study was that over-rating the healthiness of foods (i.e. an increased sense of healthiness), but not the caloric content of the preferred food items or preference for objectively-defined healthy food items, was significantly related to endogenous dorsal striatal dopamine measures. Therefore, one explanation for our findings of a significant relationship with only perceived “healthy” foods may be that foods perceived as “healthy” are more justified as preferred. This may especially be the case since our study purposely was conducted after the subjects' mealtimes when overall desire for food should be minimal. Therefore, subjects had greater preference for over-rated “healthy” foods even though they were satiated and not hungry at the time. Future studies investigating the relationship between endogenous striatal dopamine and food preferences in hungry versus sated states would further substantiate this hypothesis.

It can also be argued that health perception requires exposure and experience with food items to gain a sense of health value, and it may be the case that dietary lifestyle differences have influenced or modified underlying dorsal striatal dopamine synthesis. Furthermore, differences with familiarity of food items could have attributed to differences in food preference or over-rating of foods as healthy. However, subjects did report at the end of the task that they were familiar with all food items (see **Methods**). Although we did not investigate differences in diet, we purposely screened subjects that were not dieting at the time of the study. Additionally, all of the subjects were young (age range 19–30) without any history of eating disorders and rated themselves as in average to excellent health. We also assessed socioeconomic status, and found no influence. However, there are other environmental influences on food preferences that in addition to striatal dopamine could be explored further in future studies.

We hypothesize that the subtle individual differences in health perception may contribute to increased BMI over time, as it has been reported that minor increases in caloric intake on a daily basis (whether perceived as “healthy” or “unhealthy”) contribute to overall weight gain [32]. Although we found no relationship between BMI and health perception here, perhaps with a greater range of BMI, over-rating of the healthiness of food items may be more pronounced in higher BMI subjects. Our lack of significant findings between BMI and food-related behaviors may also suggest that endogenous striatal dopamine is more closely related to food-related behavior than BMI itself as a phenotype, since BMI is influenced by various complicated factors and may not be the best predictor of behavior or neuroimaging findings (see [10] for review). We also did not find any predictors for the change in BMI for time elapsed between PET acquisition and behavioral testing, although the change in BMI for subjects was small and not significantly different between time points. However, future studies utilizing PET FMT dopamine synthesis measures, along with food preferences and health perception measures, in a population with greater BMI fluctuation would be of great interest.

To complement previous studies that utilized PET ligands that bind dopamine receptors, we utilized a measure of dopamine synthesis capacity and show that lower dopamine synthesis in the dorsal striatum (i.e. caudate) corresponds with higher BMI. Though it should be noted, due to the cross-sectional nature of our study, we cannot definitively conclude a cause or effect relationship to lower dorsal striatal FMT dopamine synthesis values corresponding with higher BMI. However, our study used healthy-weight to moderately overweight/obese (i.e. non-morbidly obese) individuals, and therefore our results may suggest that lower dorsal striatal presynaptic dopamine measures could correspond with a propensity towards obesity. On the other hand, it may also be the case that downregulation of presynaptic dopamine in the caudate has occurred in response to moderately higher BMI, as it has been shown that dopaminergic signaling is decreased in response to overconsumption of food in animal models [5], [33], and overconsumption of food is typically associated with weight gain leading to higher BMI. Although we used individuals with a limited range of BMI in our study, perhaps viewed as a limitation of the study, we actually find the results even more compelling in that a relationship between PET FMT dopamine synthesis and BMI is present without including morbidly obese individuals. Moreover, although our sample size (n = 16) was greater than or comparable to other sample sizes in PET FMT studies ([11], [12], [15]), replication of our findings with a larger sample size and a broader range of BMI would further substantiate our results and may find greater preferences for unhealthy food items correlating with lower PET FMT dopamine synthesis values, which were not detected in our study.

In summary, although other neurotransmitter systems are involved in feeding and weight regulation [7], our study finds a role for dorsal striatal dopamine in food preferences as well as health perception of food in humans. Future prospective studies utilizing dopamine-related PET measures are of great interest to investigate how endogenous dopamine, as well individual differences in food-related behavior, might correlate with body weight fluctuation in humans.
